# Mortality rates of patients with COVID-19 in the intensive care unit: a systematic review of the emerging literature

**DOI:** 10.1186/s13054-020-03006-1

**Published:** 2020-06-04

**Authors:** Pipetius Quah, Andrew Li, Jason Phua

**Affiliations:** 1 Division of Respiratory and Critical Care Medicine, Department of Medicine, National University Hospital, National University Health System, Singapore, Singapore; 2 Fast and Chronic Programmes, Alexandra Hospital, National University Health System, Singapore, Singapore

The understanding of outcomes in the intensive care unit (ICU) for the coronavirus disease 2019 (COVID-19) remains poor. Studies have reported close to 100% mortality amongst patients requiring mechanical ventilation [1], and this together with the hypothesis that COVID-19 may not cause classic acute respiratory distress syndrome (ARDS) has led to concerns regarding the use of mechanical ventilation [2, 3]. We thus aimed to review the outcomes of ICU patients with COVID-19 from the existing literature.

We searched PubMed for studies published between Dec 1, 2019, and May 8, 2020, with at least ten ICU patients with COVID-19 and reported ICU mortality data. We excluded studies that had duplicate patients from other reports, did not provide data on ICU survival, enrolled only decedents, and excluded patients who were still hospitalised (Fig. 1 and Electronic Supplementary Material).

**Fig. 1:**
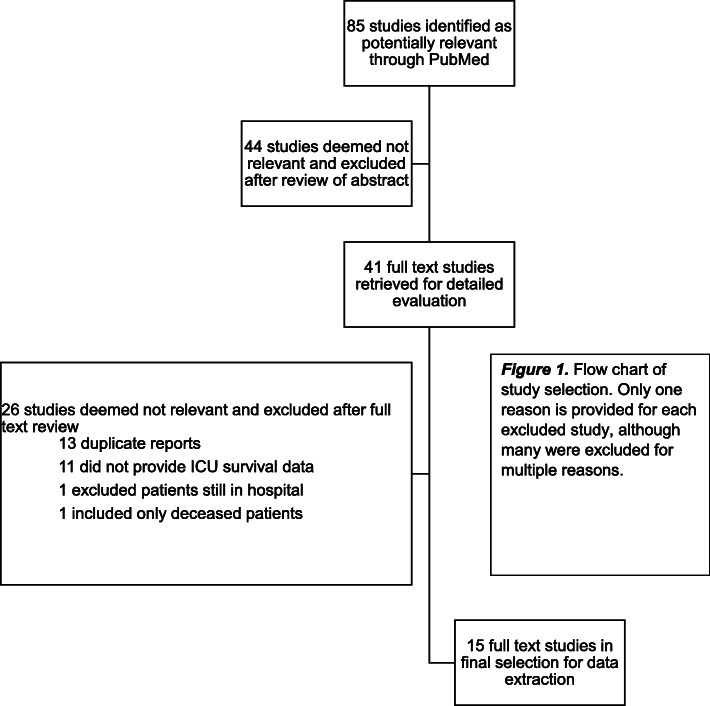
Flow chart of the study selection. Only one reason is provided for each excluded study, although many were excluded for multiple reasons

Several lessons can be surmised from Table 1, which outlines the 15 included studies conducted largely in countries worst hit by the pandemic. First, 56.1% of patients were still in the ICU at the time of study publication, and attempts to calculate mortality based on a sample of only deceased or discharged patients risk painting a skewed picture of reality [4]. Second, with the prior limitation in mind, the overall ICU mortality rate was 25.7%. In China, with 14.1% of patients still in the ICU, the mortality rate was 37.7%. These figures are not higher than the mortality rates of 35 to 45% seen in ARDS. Third, 29% of the ICU patients who died in the Chinese studies did not receive mechanical ventilation, and where systems experienced a surge of critically ill patients, up to 53.2% of patients who required ICU care were unable to receive it because of resource constraints [5]. In New York, 262 deaths occurred in hospital wards and outside the ICU, compared to 291 deaths in the ICU [4]. We hypothesise that rationing of ventilators and ICU beds in overwhelmed health systems may have resulted in attempts at postponing intubation, with a significant minority of patients received high-flow nasal cannula (13.7%) and noninvasive ventilation (11.3%) based on available data, despite uncertainty surrounding their roles.

**Table 1:** Respiratory support and outcomes for intensive care unit patients

Study	ICU sample size	Respiratory support				ICU outcomes		
		HFNC	NIV	IMV	IMV deaths	Deaths	Still in ICU	Discharged from ICUs
**China**	**517**	**81 (15.7%)**	**118 (22.8%)**	**183 (35.4%)**	**132/167 (79.0%)**	**195 (37.7%)**	**73 (14.1%)**	**249 (48.2%)**
Yang, Wuhan	52	33 (63.5%)	29 (55.8%)	22 (42.3%)	19 (86.4%)	32 (61.5%)	12 (23.1%)	8 (15.4%)
Wang, Wuhan	36	4 (11.1%)	15 (41.7%)	17 (47.2%)	6 (35.3%)	6 (16.7%)	11 (30.6%)	19 (52.8%)
Zhang, Wuhan	44	0 (0%)	27 (61.4%)	16 (36.4%)	NA	9 (20.5%)	12 (27.3%)	23 (52.3%)
Wang, Wuhan	344	35 (10.2%)	34 (9.9%)	100 (29.1%)	97 (97.0%)	133 (38.7%)	26 (7.6%)	185 (53.8%)
Zhang, Wuhan	20	0	0	20 (100%)	7 (35.0%)	12 (60%)	7 (35.0%)	1 (5.0%)
Zhou, Jiangsu	21	9 (42.9%)	13 (61.9%)	8 (38.1%)	3 (37.5%)	3 (14.3%)	5 (23.8%)	13 (61.9%)
**Italy**	**1591**	**NA**	**137 (8.6%)**	**1150 (72.3%)**	**405/1150 (35.2%)**	**405 (25.6%)**	**920 (58.2%)**	**256 (16.2%)**
Grasselli, Lombardy	1591	NA	137 (8.6%)	1150 (72.3%)	405 (35.2)	405 (25.6%)*	920 (58.2%)*	256 (16.2%)*
**USA**	**1392**	**11 (0.8%)**	**4 (0.3%)**	**1250 (89.8%)**	**305/1235 (24.7%)**	**328 (23.6%)**	**921 (66.2%)**	**143 (10.3%)**
Arentz, Washington	21	1 (4.8%)	4 (19.0%)	15 (71.4%)	NA	14 (66.7%)	5 (23.8%)	2 (9.5%)
Bhatraju, Washington	24	10 (41.7%)	0 (0%)	18 (75.0%)	12 (66.7%)	12 (50.0%)	3 (12.5%)	9 (37.5%)
Richardson, New York	1281	NA	NA	1151 (89.9%)	282 (24.5%)	291 (22.7%)	908 (70.9%)	82 (6.4%)
Ziehr, Boston	66	0	0	66 (100%)	11 (16.7%)	11 (16.7%)	5 (7.6%)	50 (75.8%)
**Spain**	**48**	**3 (6.3%)**	**0 (0%)**	**45 (93.8%)**	**14/45 (31.1%)**	**14 (29.2%)**	**21 (43.8%)**	**13 (27.1%)**
Barrasa, Vitoria	48	3 (6.3%)	0 (0%)	45 (93.8%)	14 (31.1%)	14 (29.2%)	21 (43.8%)	13 (27.1%)
**Denmark**	**17**	**0**	**0**	**17 (100%)**	**7 /17 (41.2%)**	**7 (41.2%)**	**6 (35.3%)**	**4 (23.5%)**
Pedersen, Zealand	17	0	0	17 (100%)	7 (41.2%)	7 (41.2%)	6 (35.3%)	4 (23.5%)
**Germany**	**37**	**NA**	**NA**	**NA**	**NA**	**9 (24.3%)**	**21 (56.8%)**	**7 (18.9%)**
Rieg, Freiburg	37	NA	NA	NA	NA	9 (24.3%)	21 (56.8%)	7 (18.9%)
**UK**	**196**	**NA**	**NA**	**132 (66.3%)**	**NA**	**16 (8.0%)**	**163 (81.9%)**	**17 (8.5%)**
Mahase, UK	196	NA	NA	132 (66.3%)	NA	16 (8.0%)	163 (81.9%)	17 (8.5%)
**Total**	**3798**	**95/693 (13.7%)**	**259/2284 (11.3%)**	**2645/3761 (70.3%)**	**863/2482 (34.8%)**	974/3788* (25.7%)	2125/3788* (56.1%)	689/3788* (18.2%)

Data are presented as *n* (%). *ICU* intensive care unit, *HFNC* high-flow nasal cannula, *NIV* noninvasive ventilation, *IMV* invasive mechanical ventilation, *NA* data not available. *Data on disposition available for 1581 out of 1591 patients in the study by Grasselli et al., hence the denominator for ICU outcomes is 3788 rather than 3798

We conclude that while there is a need for further studies which capture patients’ final dispositions, the current preliminary data does not suggest unusually high ICU mortality rates for COVID-19. The poor outcomes seen in various studies may be related to rationing of resources in overwhelmed ICUs.

## Supplementary information

**Additional file 1.** Electronic Supplementary Material.

## Data Availability

The datasets generated during and/or analysed during the current study are available in the PubMed repository. The full list of included studies is available in the Electronic Supplementary Data (Appendix).
