# Supplementary material for: Mortality rates of patients with COVID-19 in the intensive care unit: a systematic review of the emerging literature
Source: Crit Care. 2020 Jun 4;24:285. doi: 10.1186/s13054-020-03006-1 (PMC7271132; doi:10.1186/s13054-020-03006-1)
Supplement: Supplementary file 1 — Additional file 1. Electronic Supplementary Material. [file 13054_2020_3006_MOESM1_ESM.docx]

**Mortality rates of patients with COVID-19 in the intensive care unit:**

**A systematic review of the emerging literature**

**Electronic Supplementary Material**

**Authors:**

Pipetius Quah^1^, MRCP

Andrew Li^1^, MRCP

Jason Phua^1,2^, MRCP

^1^Division of Respiratory and Critical Care Medicine, Department of Medicine, National University Hospital, National University Health System, Singapore

^2^Fast and Chronic Programmes, Alexandra Hospital, National University Health System, Singapore

**Corresponding Author:**

Dr Pipetius Quah

Address:

Division of Respiratory and Critical Care Medicine, National University Hospital

NUHS Tower Block, Level 10

5 Lower Kent Ridge Road, Singapore 119228

Email: [pipetius_quah@nuhs.edu.sg](mailto:pipetius_quah@nuhs.edu.sg)

Telephone: +65-67795555

**Contents**

Methods: Search strategy, study selection, and data extraction: Page 3

Supplementary Figure 1: Flow chart of study selection Page 5

List of full text studies used for data extraction Page 6

List of excluded full text studies Page 10

**METHODS**

**Search strategy**

We searched PubMed using a sensitive strategy without language restrictions to identify relevant studies. The search was supplemented by reviewing references of included articles. We omitted pre-print articles.

**Study selection**

We reviewed identified articles independently and in duplicate. Full text copies of potentially relevant studies were made available to two reviewers (PQ and AL). Disagreements were resolved by the addition of a third reviewer (JP) and subsequently by consensus. We evaluated studies published between Dec 1, 2019 and May 8, 2020, using combinations of the terms “coronavirus”, “COVID-19”, “SARS-CoV-2”, “nCoV”, “intensive care”, “mechanical ventilation”, “outcomes”, and “mortality”. We retrieved studies with at least ten intensive care unit (ICU) patients with COVID-19, and which reported ICU mortality data (41 studies). We excluded studies that had duplicate patients from other reports (13 studies), did not provide data on ICU survival (i.e. discharge from the ICU, 11 studies), enrolled only decedents (1 study), and excluded patients who were still hospitalised (1 study) (appendix p4).

**Data extraction**

Two reviewers assessed methodological quality and extracted data (PQ and AL). The primary outcome was ICU mortality. This information was available in 40 of the studies. We extracted data on the ICU outcomes (death, continued hospitalisation in the ICU, and discharge from the ICU), mortality while on mechanical ventilation and the types of respiratory support of patients in the ICU.

**List of full text studies used for data extraction**

1. Arentz M, Yim E, Klaff L, Lokhandwala S, Riedo FX, Chong M, Lee M (2020) Characteristics and outcomes of 21 critically ill patients with COVID-19 in Washington State. JAMA. doi:10.1001/jama.2020.4326

2. Barrasa H, Rello J, Tejada S, Martin A, Balziskueta G, Vinuesa C, Fernandez-Miret B, Villagra A, Vallejo A, San Sebastian A, Cabanes S, Iribarren S, Fonseca F, Maynar J, Alava C-SI (2020) SARS-CoV-2 in Spanish intensive care units: early experience with 15-day survival in Vitoria. Anaesth Crit Care Pain Med. doi:10.1016/j.accpm.2020.04.001

3. Bhatraju PK, Ghassemieh BJ, Nichols M, Kim R, Jerome KR, Nalla AK, Greninger AL, Pipavath S, Wurfel MM, Evans L, Kritek PA, West TE, Luks A, Gerbino A, Dale CR, Goldman JD, O'Mahony S, Mikacenic C (2020) COVID-19 in critically ill patients in the Seattle region - case series. N Engl J Med. doi:10.1056/NEJMoa2004500

4. Grasselli G, Zangrillo A, Zanella A, Antonelli M, Cabrini L, Castelli A, Cereda D, Coluccello A, Foti G, Fumagalli R, Iotti G, Latronico N, Lorini L, Merler S, Natalini G, Piatti A, Ranieri MV, Scandroglio AM, Storti E, Cecconi M, Pesenti A, Network C-LI, Nailescu A, Corona A, Zangrillo A, Protti A, Albertin A, Forastieri Molinari A, Lombardo A, Pezzi A, Benini A, Scandroglio AM, Malara A, Castelli A, Coluccello A, Micucci A, Pesenti A, Sala A, Alborghetti A, Antonini B, Capra C, Troiano C, Roscitano C, Radrizzani D, Chiumello D, Coppini D, Guzzon D, Costantini E, Malpetti E, Zoia E, Catena E, Agosteo E, Barbara E, Beretta E, Boselli E, Storti E, Harizay F, Della Mura F, Lorini FL, Donato Sigurta F, Marino F, Mojoli F, Rasulo F, Grasselli G, Casella G, De Filippi G, Castelli G, Aldegheri G, Gallioli G, Lotti G, Albano G, Landoni G, Marino G, Vitale G, Battista Perego G, Evasi G, Citerio G, Foti G, Natalini G, Merli G, Sforzini I, Bianciardi L, Carnevale L, Grazioli L, Cabrini L, Guatteri L, Salvi L, Dei Poli M, Galletti M, Gemma M, Ranucci M, Riccio M, Borelli M, Zambon M, Subert M, Cecconi M, Mazzoni MG, Raimondi M, Panigada M, Belliato M, Bronzini N, Latronico N, Petrucci N, Belgiorno N, Tagliabue P, Cortellazzi P, Gnesin P, Grosso P, Gritti P, Perazzo P, Severgnini P, Ruggeri P, Sebastiano P, Covello RD, Fernandez-Olmos R, Fumagalli R, Keim R, Rona R, Valsecchi R, Cattaneo S, Colombo S, Cirri S, Bonazzi S, Greco S, Muttini S, Langer T, Alaimo V, Viola U (2020) Baseline characteristics and outcomes of 1591 patients infected with SARS-CoV-2 admitted to ICUs of the Lombardy region, Italy. JAMA. doi:10.1001/jama.2020.5394

5. Mahase E (2020) Covid-19: most patients require mechanical ventilation in first 24 hours of critical care. BMJ 368:m1201. doi:10.1136/bmj.m1201

6. Pedersen HP, Hildebrandt T, Poulsen A, Uslu B, Knudsen HH, Roed J, Poulsen TD, Nielsen HB (2020) Initial experiences from patients with COVID-19 on ventilatory support in Denmark. Dan Med J 67 (5)

7. Richardson S, Hirsch JS, Narasimhan M, Crawford JM, McGinn T, Davidson KW, and the Northwell C-RC, Barnaby DP, Becker LB, Chelico JD, Cohen SL, Cookingham J, Coppa K, Diefenbach MA, Dominello AJ, Duer-Hefele J, Falzon L, Gitlin J, Hajizadeh N, Harvin TG, Hirschwerk DA, Kim EJ, Kozel ZM, Marrast LM, Mogavero JN, Osorio GA, Qiu M, Zanos TP (2020) Presenting characteristics, comorbidities, and outcomes among 5700 patients hospitalized with COVID-19 in the New York City area. JAMA. doi:10.1001/jama.2020.6775

8. Rieg S, Busch HJ, Hans F, Grundmann H, Biever P, Burkle H, Hammer T, Thimme R, Kern W (2020) [COVID-19-Response - Strategies of the Task-Force Coronavirus and experiences upon implementation in the management of 115 cases at the University Medical Center Freiburg]. Dtsch Med Wochenschr. doi:10.1055/a-1147-6244

9. Wang D, Hu B, Hu C, Zhu F, Liu X, Zhang J, Wang B, Xiang H, Cheng Z, Xiong Y, Zhao Y, Li Y, Wang X, Peng Z (2020) Clinical characteristics of 138 hospitalized patients with 2019 novel coronavirus-infected pneumonia in Wuhan, China. JAMA. doi:10.1001/jama.2020.1585

10. Wang Y, Lu X, Chen H, Chen T, Su N, Huang F, Zhou J, Zhang B, Li Y, Yan F, Wang J (2020) Clinical course and outcomes of 344 intensive care patients with COVID-19. Am J Respir Crit Care Med. doi:10.1164/rccm.202003-0736LE

11. Yang X, Yu Y, Xu J, Shu H, Xia J, Liu H, Wu Y, Zhang L, Yu Z, Fang M, Yu T, Wang Y, Pan S, Zou X, Yuan S, Shang Y (2020) Clinical course and outcomes of critically ill patients with SARS-CoV-2 pneumonia in Wuhan, China: a single-centered, retrospective, observational study. Lancet Respir Med 8 (5):475-481. doi:10.1016/S2213-2600(20)30079-5

12. Zhang G, Hu C, Luo L, Fang F, Chen Y, Li J, Peng Z, Pan H (2020) Clinical features and short-term outcomes of 221 patients with COVID-19 in Wuhan, China. J Clin Virol 127:104364. doi:10.1016/j.jcv.2020.104364

13. Zhang L, Li J, Zhou M, Chen Z (2020) Summary of 20 tracheal intubation by anesthesiologists for patients with severe COVID-19 pneumonia: retrospective case series. J Anesth. doi:10.1007/s00540-020-02778-8

14. Zhou Y, Han T, Chen J, Hou C, Hua L, He S, Guo Y, Zhang S, Wang Y, Yuan J, Zhao C, Zhang J, Jia Q, Zuo X, Li J, Wang L, Cao Q, Jia E (2020) Clinical and autoimmune characteristics of severe and critical cases with COVID-19. Clin Transl Sci. doi:10.1111/cts.12805

15. Ziehr DR, Alladina J, Petri CR, Maley JH, Moskowitz A, Medoff BD, Hibbert KA, Thompson BT, Hardin CC (2020) Respiratory pathophysiology of mechanically ventilated patients with COVID-19: a cohort study. Am J Respir Crit Care Med. doi:10.1164/rccm.202004-1163LE

**List of excluded full text studies**

1. Cao B, Wang Y, Wen D, Liu W, Wang J, Fan G, Ruan L, Song B, Cai Y, Wei M, Li X, Xia J, Chen N, Xiang J, Yu T, Bai T, Xie X, Zhang L, Li C, Yuan Y, Chen H, Li H, Huang H, Tu S, Gong F, Liu Y, Wei Y, Dong C, Zhou F, Gu X, Xu J, Liu Z, Zhang Y, Li H, Shang L, Wang K, Li K, Zhou X, Dong X, Qu Z, Lu S, Hu X, Ruan S, Luo S, Wu J, Peng L, Cheng F, Pan L, Zou J, Jia C, Wang J, Liu X, Wang S, Wu X, Ge Q, He J, Zhan H, Qiu F, Guo L, Huang C, Jaki T, Hayden FG, Horby PW, Zhang D, Wang C (2020) A trial of lopinavir-ritonavir in adults hospitalized with severe COVID-19. N Engl J Med 382 (19):1787-1799. doi:10.1056/NEJMoa2001282

2. Cao J, Tu WJ, Cheng W, Yu L, Liu YK, Hu X, Liu Q (2020) Clinical features and short-term outcomes of 102 patients with corona virus disease 2019 in Wuhan, China. Clin Infect Dis. doi:10.1093/cid/ciaa243

3. Chen J, Qi T, Liu L, Ling Y, Qian Z, Li T, Li F, Xu Q, Zhang Y, Xu S, Song Z, Zeng Y, Shen Y, Shi Y, Zhu T, Lu H (2020) Clinical progression of patients with COVID-19 in Shanghai, China. J Infect 80 (5):e1-e6. doi:10.1016/j.jinf.2020.03.004

4. Chen N, Zhou M, Dong X, Qu J, Gong F, Han Y, Qiu Y, Wang J, Liu Y, Wei Y, Xia J, Yu T, Zhang X, Zhang L (2020) Epidemiological and clinical characteristics of 99 cases of 2019 novel coronavirus pneumonia in Wuhan, China: a descriptive study. Lancet 395 (10223):507-513. doi:10.1016/S0140-6736(20)30211-7

5. Chen T, Dai Z, Mo P, Li X, Ma Z, Song S, Chen X, Luo M, Liang K, Gao S, Zhang Y, Deng L, Xiong Y (2020) Clinical characteristics and outcomes of older patients with coronavirus disease 2019 (COVID-19) in Wuhan, China (2019): a single-centered, retrospective study. J Gerontol A Biol Sci Med Sci. doi:10.1093/gerona/glaa089

6. Du RH, Liu LM, Yin W, Wang W, Guan LL, Yuan ML, Li YL, Hu Y, Li XY, Sun B, Peng P, Shi HZ (2020) Hospitalization and critical care of 109 decedents with COVID-19 pneumonia in Wuhan, China. Ann Am Thorac Soc. doi:10.1513/AnnalsATS.202003-225OC

7. Geleris J, Sun Y, Platt J, Zucker J, Baldwin M, Hripcsak G, Labella A, Manson D, Kubin C, Barr RG, Sobieszczyk ME, Schluger NW (2020) Observational study of hydroxychloroquine in hospitalized patients with COVID-19. N Engl J Med. doi:10.1056/NEJMoa2012410

8. Gouel-Cheron A, Couffignal C, Elmaleh Y, Kantor E, Montravers P (2020) Preliminary observations of anaesthesia ventilators use for prolonged mechanical ventilation in intensive care unit patients during the COVID-19 pandemic. Anaesth Crit Care Pain Med. doi:10.1016/j.accpm.2020.04.009

9. Grein J, Ohmagari N, Shin D, Diaz G, Asperges E, Castagna A, Feldt T, Green G, Green ML, Lescure FX, Nicastri E, Oda R, Yo K, Quiros-Roldan E, Studemeister A, Redinski J, Ahmed S, Bernett J, Chelliah D, Chen D, Chihara S, Cohen SH, Cunningham J, D'Arminio Monforte A, Ismail S, Kato H, Lapadula G, L'Her E, Maeno T, Majumder S, Massari M, Mora-Rillo M, Mutoh Y, Nguyen D, Verweij E, Zoufaly A, Osinusi AO, DeZure A, Zhao Y, Zhong L, Chokkalingam A, Elboudwarej E, Telep L, Timbs L, Henne I, Sellers S, Cao H, Tan SK, Winterbourne L, Desai P, Mera R, Gaggar A, Myers RP, Brainard DM, Childs R, Flanigan T (2020) Compassionate use of Remdesivir for patients with severe COVID-19. N Engl J Med. doi:10.1056/NEJMoa2007016

10. Guan WJ, Ni ZY, Hu Y, Liang WH, Ou CQ, He JX, Liu L, Shan H, Lei CL, Hui DSC, Du B, Li LJ, Zeng G, Yuen KY, Chen RC, Tang CL, Wang T, Chen PY, Xiang J, Li SY, Wang JL, Liang ZJ, Peng YX, Wei L, Liu Y, Hu YH, Peng P, Wang JM, Liu JY, Chen Z, Li G, Zheng ZJ, Qiu SQ, Luo J, Ye CJ, Zhu SY, Zhong NS, China Medical Treatment Expert Group for C (2020) Clinical characteristics of coronavirus disease 2019 in China. N Engl J Med 382 (18):1708-1720. doi:10.1056/NEJMoa2002032

11. Huang C, Wang Y, Li X, Ren L, Zhao J, Hu Y, Zhang L, Fan G, Xu J, Gu X, Cheng Z, Yu T, Xia J, Wei Y, Wu W, Xie X, Yin W, Li H, Liu M, Xiao Y, Gao H, Guo L, Xie J, Wang G, Jiang R, Gao Z, Jin Q, Wang J, Cao B (2020) Clinical features of patients infected with 2019 novel coronavirus in Wuhan, China. Lancet 395 (10223):497-506. doi:10.1016/S0140-6736(20)30183-5

12. Jacobs JP, Stammers AH, St Louis J, Hayanga JWA, Firstenberg MS, Mongero LB, Tesdahl EA, Rajagopal K, Cheema FH, Coley T, Badhwar V, Sestokas AK, Slepian MJ (2020) Extracorporeal membrane oxygenation in the treatment of severe pulmonary and cardiac compromise in COVID-19: experience with 32 patients. ASAIO J. doi:10.1097/MAT.0000000000001185

13. Li Y, Hu Y, Yu J, Ma T (2020) Retrospective analysis of laboratory testing in 54 patients with severe- or critical-type 2019 novel coronavirus pneumonia. Lab Invest. doi:10.1038/s41374-020-0431-6

14. Marullo AG, Cavarretta E, Biondi-Zoccai G, Mancone M, Peruzzi M, Piscioneri F, Sartini P, Versaci F, Morelli A, Miraldi F, Frati G (2020) Extracorporeal membrane oxygenation for critically ill patients with coronavirus-associated disease 2019: an updated perspective of the European experience. Minerva Cardioangiol. doi:10.23736/S0026-4725.20.05328-1

15. Mehra MR, Desai SS, Kuy S, Henry TD, Patel AN (2020) Cardiovascular Disease, drug therapy, and mortality in Covid-19. N Engl J Med. doi:10.1056/NEJMoa2007621

16. Mehta V, Goel S, Kabarriti R, Cole D, Goldfinger M, Acuna-Villaorduna A, Pradhan K, Thota R, Reissman S, Sparano JA, Gartrell BA, Smith RV, Ohri N, Garg M, Racine AD, Kalnicki S, Perez-Soler R, Halmos B, Verma A (2020) Case fatality rate of cancer patients with COVID-19 in a New York hospital system. Cancer Discov. doi:10.1158/2159-8290.CD-20-0516

17. Miyashita H, Mikami T, Chopra N, Yamada T, Chernyavsky S, Rizk D, Cruz C (2020) Do patients with cancer have a poorer prognosis of COVID-19? An experience in New York City. Ann Oncol. doi:10.1016/j.annonc.2020.04.006

18. Peng YD, Meng K, Guan HQ, Leng L, Zhu RR, Wang BY, He MA, Cheng LX, Huang K, Zeng QT (2020) [Clinical characteristics and outcomes of 112 cardiovascular disease patients infected by 2019-nCoV]. Zhonghua Xin Xue Guan Bing Za Zhi 48 (0):E004. doi:10.3760/cma.j.cn112148-20200220-00105

19. Ruan Q, Yang K, Wang W, Jiang L, Song J (2020) Clinical predictors of mortality due to COVID-19 based on an analysis of data of 150 patients from Wuhan, China. Intensive Care Med 46 (5):846-848. doi:10.1007/s00134-020-05991-x

20. Wang D, Yin Y, Hu C, Liu X, Zhang X, Zhou S, Jian M, Xu H, Prowle J, Hu B, Li Y, Peng Z (2020) Clinical course and outcome of 107 patients infected with the novel coronavirus, SARS-CoV-2, discharged from two hospitals in Wuhan, China. Crit Care 24 (1):188. doi:10.1186/s13054-020-02895-6

21. Wang K, Zhang Z, Yu M, Tao Y, Xie M (2020) 15-day mortality and associated risk factors for hospitalized patients with COVID-19 in Wuhan, China: an ambispective observational cohort study. Intensive Care Med. doi:10.1007/s00134-020-06047-w

22. Wang R, Pan M, Zhang X, Fan X, Han M, Zhao F, Miao M, Xu J, Guan M, Deng X, Chen X, Shen L (2020) Epidemiological and clinical features of 125 Hospitalized Patients with COVID-19 in Fuyang, Anhui, China. Int J Infect Dis. doi:10.1016/j.ijid.2020.03.070

23. Wu C, Chen X, Cai Y, Xia J, Zhou X, Xu S, Huang H, Zhang L, Zhou X, Du C, Zhang Y, Song J, Wang S, Chao Y, Yang Z, Xu J, Zhou X, Chen D, Xiong W, Xu L, Zhou F, Jiang J, Bai C, Zheng J, Song Y (2020) Risk factors associated with acute respiratory distress syndrome and death in patients with coronavirus disease 2019 pneumonia in Wuhan, China. JAMA Intern Med. doi:10.1001/jamainternmed.2020.0994

24. Zangrillo A, Beretta L, Scandroglio AM, Monti G, Fominskiy E, Colombo S, Morselli F, Belletti A, Silvani P, Crivellari M, Monaco F, Azzolini ML, Reineke R, Nardelli P, Sartorelli M, Votta CD, Ruggeri A, Ciceri F, De Cobelli F, Tresoldi M, Dagna L, Rovere-Querini P, Serpa Neto A, Bellomo R, Landoni G, Group CO-BS (2020) Characteristics, treatment, outcomes and cause of death of invasively ventilated patients with COVID-19 ARDS in Milan, Italy. Crit Care Resusc

25. Zhang J, Liu P, Wang M, Wang J, Chen J, Yuan W, Li M, Xie Z, Dong W, Li H, Zhao Y, Wan L, Chu T, Wang L, Zhang H, Tao T, Ma J (2020) The clinical data from 19 critically ill patients with coronavirus disease 2019: a single-centered, retrospective, observational study. Z Gesundh Wiss:1-4. doi:10.1007/s10389-020-01291-2

26. Zhou F, Yu T, Du R, Fan G, Liu Y, Liu Z, Xiang J, Wang Y, Song B, Gu X, Guan L, Wei Y, Li H, Wu X, Xu J, Tu S, Zhang Y, Chen H, Cao B (2020) Clinical course and risk factors for mortality of adult inpatients with COVID-19 in Wuhan, China: a retrospective cohort study. Lancet 395 (10229):1054-1062. doi:10.1016/S0140-6736(20)30566-3
